# Development and validation of a nomogram to predict allograft survival after pediatric liver transplantation

**DOI:** 10.1007/s12519-023-00766-y

**Published:** 2023-10-24

**Authors:** Guang-Xiang Gu, Shu-Ting Pan, Yi-Chen Fan, Chen Chen, Qiang Xia

**Affiliations:** 1 Department of Liver Surgery, Renji Hospital, Shanghai Jiaotong University School of Medicine, No. 160 Pujian Road, Pudong New District, Shanghai, 200128 China; 2 Department of Liver Transplantation, Sun Yet-Sen Memorial Hospital, Sun Yat-Sen University, No.107 Yanjiang West Road, Guangzhou, 510080 China; 3 Clinical Center for Investigation, Renji Hospital, Shanghai Jiaotong University School of Medicine, Shanghai, China; 4 Department of Anesthesiology, Renji Hospital, Shanghai Jiaotong University School of Medicine, Shanghai, China

**Keywords:** Allograft survival, Nomogram, Pediatric liver transplantation

## Abstract

**Background:**

Liver transplantation is the main treatment for cholestatic liver disease and some metabolic liver diseases in children. However, no accurate prediction model to determine the survival probability of grafts prior to surgery exists. This study aimed to develop an effective prognostic model for allograft survival after pediatric liver transplantation.

**Methods:**

This retrospective cohort study included 2032 patients who underwent pediatric liver transplantation between January 1, 2006, and January 1, 2020. A nomogram was developed using Cox regression and validated based on bootstrap sampling. Predictive and discriminatory accuracies were determined using the concordance index and visualized using calibration curves; net benefits were calculated for model comparison. An online Shiny application was developed for easy access to the model.

**Results:**

Multivariable analysis demonstrated that preoperative diagnosis, recipient age, body weight, graft type, preoperative total bilirubin, interleukin-1β, portal venous blood flow direction, spleen thickness, and the presence of heart disease and cholangitis were independent factors for survival, all of which were selected in the nomogram. Calibration of the nomogram indicated that the 1-, 3-, and 5-year predicted survival rates agreed with the actual survival rate. The concordance indices for graft survival at 1, 3, and 5 years were 0.776, 0.757, and 0.753, respectively, which were significantly higher than those of the Pediatric End-Stage Liver Disease and Child–Pugh scoring systems. The allograft dysfunction risk of a recipient could be easily predicted using the following URL: https://aspelt.shinyapps.io/ASPELT//

**Conclusion:**

The allograft survival after pediatric liver transplantation (ASPELT) score model can effectively predict the graft survival rate after liver transplantation in children, providing a simple and convenient evaluation method for clinicians and patients.

**Supplementary Information:**

The online version contains supplementary material available at 10.1007/s12519-023-00766-y.

## Introduction

Pediatric liver transplantation (LT) is the main treatment for cholestatic liver disease and some metabolic liver diseases in children [1]. Since the successful implementation of the first LT by Starzl et al. [2] in 1967, adult and pediatric LTs have been developing continuously. Currently, pediatric LT is a mature treatment for children with end-stage liver diseases, such as acute liver failure, autoimmune diseases, cholestasis, metabolic or genetic diseases, and oncologic and vascular liver diseases [3, 4]. With the development of immunosuppressive drugs and postoperative care, the mortality rate of recipients and grafts has decreased every year. The 1-year and 5-year survival rates after pediatric LT have been reported to exceed 90% and 85%, respectively [4]. However, despite these improvements in treatment techniques, graft dysfunction still poses a significant challenge, directly impacting recipient prognosis. Graft dysfunction in children might result in secondary transplantation or even death [5, 6]. The main causes of dysfunction include chronic rejection, biliary tract or vascular complications, and inflammation or infection [7–9].

There are a few clinical methods to predict the pediatric LT survival rate at present, such as Pediatric risk of mortality (PRISM) III, Survival Outcomes Following Pediatric Liver Transplant (SOFT), Pediatric End-Stage Liver Disease (PELD), and Child–Pugh [8, 10], with the last two being the most widely used scoring systems for liver diseases. A higher score in each of these methods indicates a lower prognosis for patients. PELD scores are primarily used in patients younger than 12 years of age, whereas Child–Pugh scores determine the prognosis of patients with liver cirrhosis. However, these scoring tools are not specifically designed for predicting graft survival, although they have been applied to evaluate allograft outcomes in previous studies [11–13]. Unfortunately, there is a lack of comprehensive studies analyzing the risk factors associated with allograft outcomes, and as of now, an accurate prediction model for assessing the survival probability of grafts prior to surgery remains elusive. Therefore, the main purposes of this study were to determine the risk factors affecting graft survival and to construct a relatively accurate predictive index using a nomogram to predict allograft survival after pediatric liver transplantation (ASPELT).

## Methods

### Study population and data acquisition

We retrospectively analyzed all the data of children younger than 12 years of age who had undergone LT at the liver surgery department in Renji Hospital affiliated with the Shanghai Jiaotong University School of Medicine from 2006 to 2019. The study protocol conformed to the ethical guidelines of the 1975 Declaration of Helsinki, as reflected in a priori approval by the institution’s human research committee (Ethics Committee of Renji Hospital No: KY2020-064). Informed consent to participate in the study was obtained from their parent or legal guardian.

Inclusion and exclusion criteria were applied during the data selection process. Children who met the following criteria were included in the study: age younger than 12 years and underwent liver transplantation at Renji Hospital between 2006 and 2019. Children who met any of the following criteria were excluded from the study: foreign children, orphans, and inadequate follow-up data.

Preoperatively, a thorough and detailed physical examination; laboratory tests including liver and kidney function tests, coagulation profiles, and interleukin (IL)-1β levels; and ultrasound examination and computed tomography were performed routinely before surgery to completely assess the preoperative condition of the recipients and determine the extent of any complications and to determine the PELD and Child–Pugh scores.

All patients were closely monitored in the intensive care unit after surgery. Blood gas, coagulation profile, and liver function tests were assessed daily. Patients were transferred to the general ward after becoming stable. The dosage of the immunosuppressant was adjusted to maintain a safe upper level according to its blood concentration. After discharge, all patients were followed up regularly at the outpatient clinic, and the medication plan was adjusted using a standard protocol.

### Study design

Overall, 23 candidate predictors, consisting of 20 indicators of recipients’ characteristics and 3 of donor characteristics, were analyzed during the training of the prediction model. The recipients’ characteristics were as follows: three physical features, namely, age, body weight, and growth failure; diagnosis; presence of any heart disease, portal hypertension, gastrointestinal bleeding, cholangitis, and ascites; preoperative levels of albumin (g/dL), total bilirubin (mg/dL), prothrombin time (s), international normalized ratio (INR), and IL-1β (pg/mL) as per the results of laboratory tests; perioperative features including direction of the portal vein flow, spleen thickness (mm), spleen diameter (mm), surgical method, graft-to-recipient weight ratio; and the year of transplantation. For donor characteristics, body mass index (BMI), relationships to recipients, and ABO compatibility were considered.

The outcome of our prediction model was graft survival, which was defined as the time from transplantation to graft dysfunction.

### Statistical analysis

Baseline characteristics were analyzed with descriptive statistics. Continuous variables were described as the mean ± standard deviation, and frequencies and percentages are used to describe categorical variables. Continuous predictors were categorized according to their optimal cutoff points that maximized the sum of sensitivity and specificity in the receiver operating characteristic (ROC) curve analyses. All covariates listed in the previous chapter were included in a univariable analysis using proportional hazards regression, and covariates with less significance (i.e., *P* value > 0.1) or multicollinearity were excluded from multivariable analysis. Before entering into the multivariable analysis, multiple imputation was implemented for covariates with missing data, and imputation methods were selected based on variable characteristics.

The prediction model was developed for 1-year, 3-year, and 5-year graft survival. Predictors were selected according to the results of multivariable analyses. In addition, the Akaike information criterion, C-index, and area under the ROC curve (AUC) were considered for determining our final predictive model. For internal validation, bootstrapping was conducted, and calibration plots were created to compare the predicted survival probabilities and actual probabilities. Model visualization was achieved using a nomogram, which explains the method of calculating predicted probabilities. To evaluate the model performance and compare it with that of the other existing and well-known scoring systems, such as PELD scores and Child–Pugh, net benefits were calculated and illustrated using decision curves. Moreover, an AUC with precision [i.e., 95% confidence interval (CI)] was generated for all models using nonparametric inverse probability weighting estimation and bootstrapping. All analyses were performed using open-source software *R* version 4.0. A *P* value of 0.05 (two-tailed) was considered to indicate statistical significance.

### Application development

An interactive web application was built and published based on the *R* package Shiny to allow easy access to the prediction model. Prediction results can be obtained after inputting patient data.

## Results

### Clinical characteristics

A total of 2080 pediatric patients under the age of 12 underwent liver transplantation (LT) at the Liver Surgery Department of Renji Hospital, which is affiliated with the Shanghai Jiaotong University School of Medicine, between the years 2006 and 2019. To enhance the reliability of our follow-up data, we excluded information from 38 foreign pediatric patients and 10 orphaned children. Consequently, our analysis was based on a cohort of 2032 pediatric patients.

**Fig. 1:**
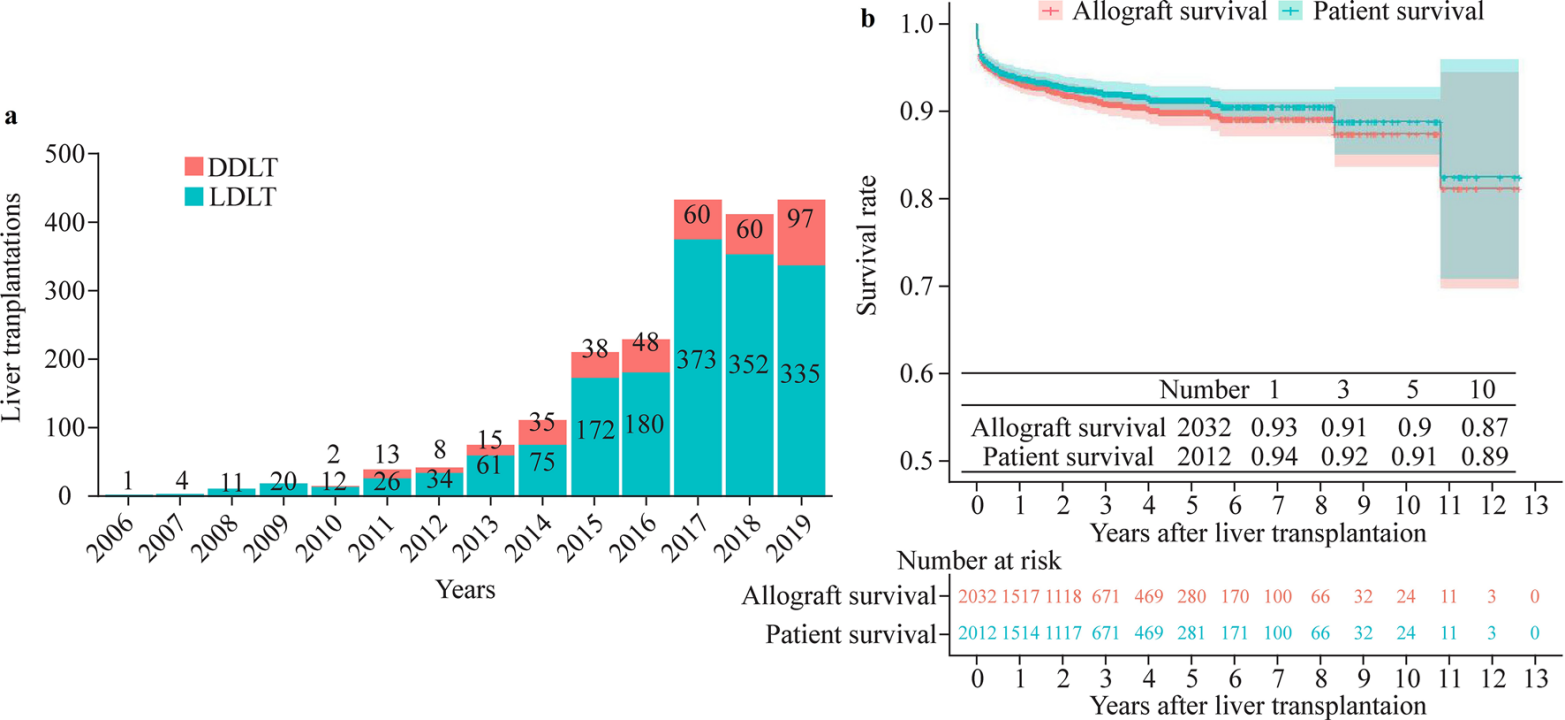
Number of pediatric liver transplantations from 2006 to 2019 at Renji Hospital and total survival rate for the allograft and patient using the Kaplan–Meier curve. The y-axis indicates the graft survival rate, and the x-axis indicates the number of years after liver transplantation (**a**). Percentages of recipient survival among the total recipients at different years are shown in the table in the middle (**b**). *DDLT* deceased donor liver transplantation, *LDLT* living donor liver transplantation

Since 2006, the number of pediatric LTs performed at Renji Hospital has been increasing year by year, especially for living donor LT. From 2017 to 2019, the number of pediatric LTs remained at approximately 400 cases annually, and the number of living donor LT cases increased much more dramatically than the number of deceased donor LT cases over the years (Fig. 1a). Supplementary Fig. 1 depicts the geographic distribution of all recipients who have undergone LT at our center.

The details of the donor and recipient characteristics are summarized in Table 1. This study included 2032 pediatric LT recipients with a median [interquartile range (IQR)] age of 8 months (6–17 months) and body weight of 7.7 kg (6.6–10 kg); there were 1067 (52.50%) girls and 965 (47.50%) boys. Overall, 211 (10.40%) patients were assessed as having comorbid heart disease; 728 (35.80%) had portal hypertension; 244 (12.00%) experienced gastrointestinal bleeding; 579 (28.50%) had cholangitis, and 1131 (55.65%) were diagnosed with ascites. According to ultrasound results, the direction of the portal vein flow was toward-liver in 1413 cases (69.5%) and ex-liver in 332 cases (16.4%), with data missing for the remaining 287 cases (14.1%). The operation methods recorded included 100 cases of split LT (4.9%), 276 cases of orthotopic LT (13.6%), and 1656 cases of living donor LT (81.5%). The median (IQR) follow-up time was 2.69 years (1.45–4.27), and the total number of deaths recorded until 2019 was 170 (8.3%).

**Table 1:** Donor and recipient characteristics for pediatric patients undergoing liver transplantation

Variables	*n* or median		% or IQR
*Total number*	2032		
Recipient demographics			
Age (mon)	8		6.00–17.00
Sex			
F	1067		52.50%
M	965		47.50%
Body weight (kg)	7.7		6.60–10.00
Height (cm)	67		64.00–76.00
Blood type			
A	628		30.90%
AB	178		8.80%
B	565		27.80%
O	661		32.50%
CYP			
AA	136		6.70%
AG	715		35.20%
GG	914		45.00%
Missing	267		13.10%
Growth failure			
No	740		36.40%
Yes	1292		63.60%
History of heart disease			
Yes	211		10.40%
No	1577		77.60%
Missing	244		12.00%
Portal hypertension			
Yes	728		35.80%
No	1004		49.40%
Missing	300		14.80%
Gastrointestinal bleeding			
Yes	244		12.00%
No	1478		72.70%
Missing	310		15.30%
Cholangitis			
Yes	579		28.50%
No	1158		57.00%
Missing	295		14.50%
Ascites			
Yes	1131		55.70%
No	678		33.40%
Missing	223		11.00%
Albumin (g/dL)	3.48		3.09–3.90
Bilirubin (mg/dL)	12.9		3.40–20.00
Prothrombin time (s)	14.7		12.50–18.90
INR	1.3		1.10–1.66
Spleen thickness (mm)	28		25.00–34.00
Spleen long diameter (mm)	90		78.00–96.00
Direction of portal vein flow			
Toward-liver	1413		69.50%
Ex-liver	332		16.40%
Missing	287		14.10%
PELD	19		11.00–27.00
Child–Pugh score	9		7.00–10.00
Child–Pugh			
A	304		15.00%
B	933		45.90%
C	795		39.10%
Donor demographics			
Age (y)		29	25.00–34.00
Sex			
F		1091	53.70%
M		941	46.30%
Height (cm)		161	150.00–170.00
Body weight (kg)		57	46.00–65.00
BMI		21.53	19.72–23.71
Blood type			
A		581	28.60%
AB		100	4.90%
B		548	27.00%
O		802	39.50%
CYP			
AA		127	6.20%
AG		608	29.90%
GG		742	36.50%
Missing		555	27.30%
Relationship between donor and recipient			
Relationships to recipients			
Father to daughter		339	16.70%
Father to son		325	16.00%
Mother to daughter		451	22.20%
Mother to son		469	23.10%
Grandparents to grandchildren		52	2.60%
Others		19	0.90%
DDLT		377	18.60%
ABO compatibility			
Compatible		404	19.90%
Identical		1403	69.00%
Incompatible		225	11.10%
Gender mismatch			
Female to female		563	27.70%
Female to male		528	26.00%
Male to female		504	24.80%
Male to male		437	21.50%
CYP compatibility			
No		569	28.00%
Yes		866	42.60%
Missing		597	29.40%
Operative characteristics			
Operative years			
2006–2009		36	1.80%
2010–2014		281	13.80%
2015–2019		1715	84.40%
Operative type			
Split liver transplantation		100	4.90%
Orthotopic liver transplantation		276	13.60%
Living donor liver transplantation		1656	81.50%
Graft type			
Left lateral segment		1458	71.80%
Reduced left lateral segment		174	8.60%
Extended left lateral segment		9	0.40%
Left lobe		92	4.50%
Right lateral segment		5	0.20%
Right lobe		18	0.90%
Whole liver		276	13.60%
RBC transfusion (units)		1	1.00–2.00
Graft weight (g)		255	220.00–300.00
GRWR			
≥ 4.16		331	16.30%
< 4.16		1701	83.70%
Post-transplant characteristics			
ICU stay (d)		5	4.00–6.00
Follow up (y)		2.69	1.45–4.27
Death		170	8.30%
Immunosuppression drug			
Cyclosporine		282	13.90%
Tacrolimus		1750	86.10%
Mycophenolate mofetil			
Yes		1287	63.30%
No		745	36.70%

*OR* odds ratio, *IQR* interquartile range, *RBC* red blood cell, *GRWR* graft-to-recipient weight ratio, *ICU* intensive care unit, *F* female, *M* male, CYP, Cytochrome P450 proteins, *INR* international normalized ratio, *PELD* Pediatric End-Stage Liver Disease, *DDLT* deceased donor liver transplantation, *BMI* body mass index

The indications for pediatric LT are listed in Supplementary Table 1. Among the 2032 cases, there were 1801 cases of cholestatic liver disease (88.6%) and 1647 cases of biliary atresia (81.1%). There were 154 cases of metabolic diseases (7.6%), 28 cases of tumor diseases (1.4%), 24 cases of retransplantation, 13 cases of acute liver failure, and 12 cases of vascular diseases. All patients received immunosuppressive therapy following the operation, with cyclosporine administered in 282 cases and tacrolimus in 1750 cases.

### Postoperative prognosis and univariable and multivariable Cox regression analyses

Among the 2032 patients who underwent pediatric LT, the 1-, 3-, 5-, and 10-year graft survival rates were 93.3%, 90.9%, 89.9%, and 87.3%, respectively. The 1-, 3-, 5-, and 10-year overall survival rates were 94%, 92%, 91%, and 89%, respectively (Fig. 1b).

The results of the univariable and multivariable Cox regression analyses are shown in Table 2. According to the univariable analysis, weight, age, diagnosis, operation type, and all five preoperative comorbidities were selected for the multivariable analysis. Moreover, preoperative laboratory measurements, including serum albumin, serum total bilirubin, prothrombin time, INR, and IL-1β were screened. Other features, including the portal vein flow direction, spleen thickness and diameter, and graft-to-recipient weight ratio along with the BMI of the donors, were also included as independent risk factors in the following analysis.

**Table 2:** Univariable and multivariable Cox regression analyses for predicting allograft survival after pediatric liver transplantation

Variables	Univariable			Multivariable		
	HR	95% CI	*P*-value	HR	95% CI	*P*-value
Recipient growth failure						
No	REF					
Yes	1.23	0.88, 1.69	0.215			
Recipient body weight						
≥ 7.2 kg	REF			REF		
< 7.2 kg	1.37	1.01, 1.85	0.041	1.60	1.08, 2.37	0.019
Recipient age						
6 mon–1 y	REF			REF		
< 6 mon	1.00	0.63, 1.57	0.992	1.21	0.75, 1.94	0.428
≥ 10 y	1.86	0.96, 3.57	0.063	2.69	1.18, 6.09	0.018
1–5 y	0.88	0.6, 1.29	0.524	1.17	0.72, 1.88	0.512
5–10 y	0.81	0.41, 1.61	0.556	1.36	0.6, 3.06	0.464
Heart disease						
No	REF			REF		
Yes	4.00	2.45, 6.51	< 0.001	2.14	1.51, 3.02	< 0.001
Portal hypertension						
No	REF			REF		
Yes	3.11	1.66, 5.8	< 0.001	1.21	0.85, 1.71	0.273
Gastrointestinal bleeding						
No	REF			REF		
Yes	3.94	1.82, 8.49	< 0.001	1.50	1, 2.24	0.046
Cholangitis						
No	REF			REF		
Yes	3.70	1.93, 7.05	< 0.001	1.85	1.35, 2.54	< 0.001
Ascites						
No	REF			REF		
Yes	7.00	3.04, 16	< 0.001	1.32	0.88, 1.96	0.174
Recipient albumin						
≥ 3.0 g/dL	REF			REF		
< 3.0 g/dL	1.64	1.18, 2.28	0.003	1.27	0.9, 1.78	0.168
Recipient total bilirubin						
< 5.3 mg/dL	REF			REF		
≥ 5.3 mg/dL	2.14	1.41, 3.22	< 0.001	2.35	1.41, 3.88	0.001
Recipient prothrombin time						
< 14.4 s	REF			REF		
≥ 14.4 s	1.38	1.01, 1.88	0.044	1.02	0.71, 1.44	0.931
Recipient INR						
Below 2.21	REF			REF		
Above 2.21	1.47	0.98, 2.21	0.062	0.76	0.48, 1.17	0.211
IL-1β						
< 7.54	REF			REF		
≥ 7.54	1.63	1.03, 4.93	0.0421	1.30	0.95, 1.77	0.077
Direction of portal vein flow						
Toward-liver	REF			REF		
Ex-liver	2.16	1.51, 3.07	< 0.001	1.70	1.19, 2.41	0.003
Recipient spleen thickness						
< 27 mm	REF			REF		
≥ 27 mm	1.53	1.04, 2.25	0.03	1.51	0.96, 2.36	0.075
Recipient spleen long diameter						
< 86 mm	REF			REF		
≥ 86 mm	1.51	1.03, 2.21	0.035	0.84	0.53, 1.31	0.437
Diagnosis						
Cholestatic liver disease	REF			REF		
Metabolic liver disease	0.48	0.21, 1.08	0.075	1.54	0.59, 3.99	0.377
Re-transplantation	4.48	0.34, 2.53	< 0.001	1.54	0.52, 4.52	0.43
Others	0.94	2.09, 9.57	0.898	3.34	1.39, 7.97	0.007
Donor BMI	0.95	0.91, 0.98	0.003	0.99	0.96, 1.01	0.337
ABO compatibility						
Identical	REF					
Compatible	1.04	0.71, 1.52	0.83			
Incompatible	1.27	0.79, 2.03	0.308			
Relationships to recipients						
Mother to daughter	REF					
Others	2.08	1.32, 3.24	0.001			
Mother to son	1.14	0.69, 1.84	0.609			
Father to daughter	1.25	0.74, 2.08	0.404			
Father to son	0.76	0.41, 1.39	0.374			
Grandparents to grandchildren	1.55	0.64, 3.73	0.325			
Operative time						
2006–2009	REF					
2010–2014	0.38	0.2, 0.71	0.003			
2015–2019	0.23	0.12, 0.41	< 0.001			
Surgical method						
Living donor liver transplantation	REF			REF		
Orthotopic liver transplantation	1.67	1.13, 2.45	0.009	1.13	0.49, 2.58	0.781
Split liver transplantation	2.00	1.14, 3.48	0.014	1.80	0.99, 3.26	0.049
GRWR						
< 4.16	REF			REF		
≥ 4.16	1.58	1.1, 2.26	0.013	1.25	0.83, 1.87	0.288

*REF* reference, *HR* hazard ratio, *CI* confidence interval, *INR* international normalized ratio, *IL* interleukin, *BMI* body mass index, *GRWR* graft-to-recipient weight ratio

The adjusted odds ratios and their precision indicated that lower body weight (< 7.2 kg), age ≥ 10 years, heart disease, cholangitis, direction of portal vein flow, spleen thickness (≥ 27 mm), retransplantation, split-liver transplantation, and higher levels of total bilirubin (≥ 5.3 mg/dL) significantly increased the risk of allograft dysfunction. The survival curves of significant covariates in each group are demonstrated in Supplementary Fig. 2, panels A-I.

### Development and validation of the allograft survival after pediatric liver transplantation nomogram

**Fig. 2:**
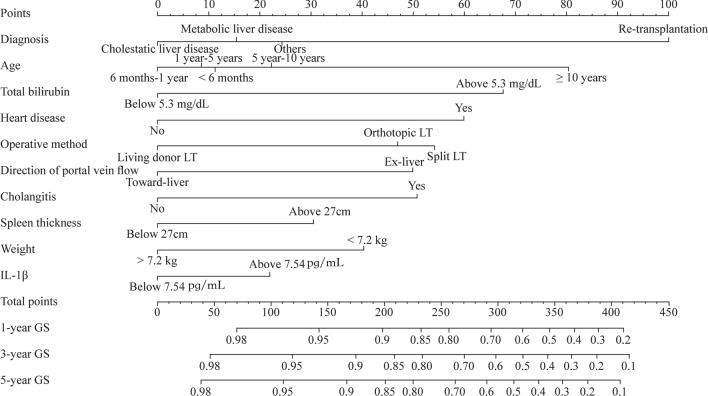
Nomogram predicting graft survival after a pediatric liver transplantation. To determine the number of points received for each level, a line is drawn upward on each variable axis. Points from every categories were summed together to acquire a total point, which will be marked on the “Total Points” axis. From each point, a line is drawn downward to the survival axes to determine the likelihood of 1-, 3-, or 5-year survival of an allograft. *IL* interleukin, *GS* graft survival, *LT* liver transplantation

Based on the multivariable Cox regression results listed in the previous section and combined with a stepwise model selection and clinical consideration, 10 variables were selected and used to construct a visualized nomogram model (Fig. 2) to predict the survival rate of allografts. Considering that IL-1β is not a routine preoperative test in some transplantation centers, we also created a model without IL-1β. Each variable has a corresponding score on the point scale axis of the nomogram. The scores of the different variables are presented in Supplementary Table 2. By adding the scores of different variables, the total score of ASPELT can be easily calculated, and the survival probability of a graft can be subsequently determined. The C-indices of the prediction nomogram, ASPELT, for predicting graft survival at 1, 3, and 5 years were 0.776, 0.757, and 0.753, respectively, whereas those of the version without IL-1β (i.e., ASPELT/IL-1β) were 0.774, 0.751, and 0.749, respectively (Supplementary Table 3).

**Fig. 3:**
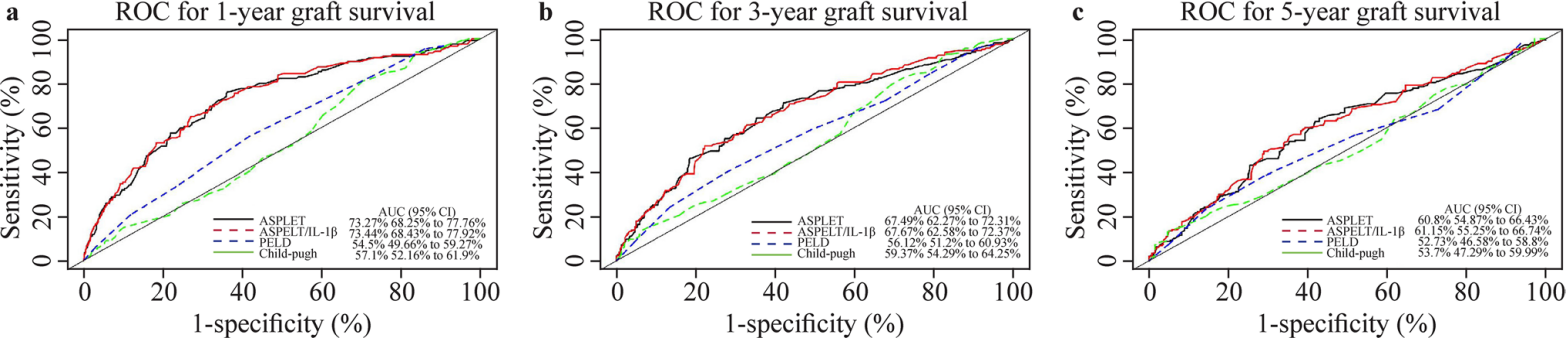
ROC curves for ASPELT, ASPELT/IL-1β, PELD, and Child–Pugh scores. Curves present the scores at (**a**) 1 year, (**b**) 3 years, and (**c**) 5 years posttransplantation. *ROC* receiver operating characteristic, *ASPELT* allograft survival after pediatric liver transplantation, *IL* interleukin, *PELD* Pediatric End-Stage Liver Disease, *AUC* area under the receiver operating characteristic curve, *CI* confidence interval

**Fig. 4:**
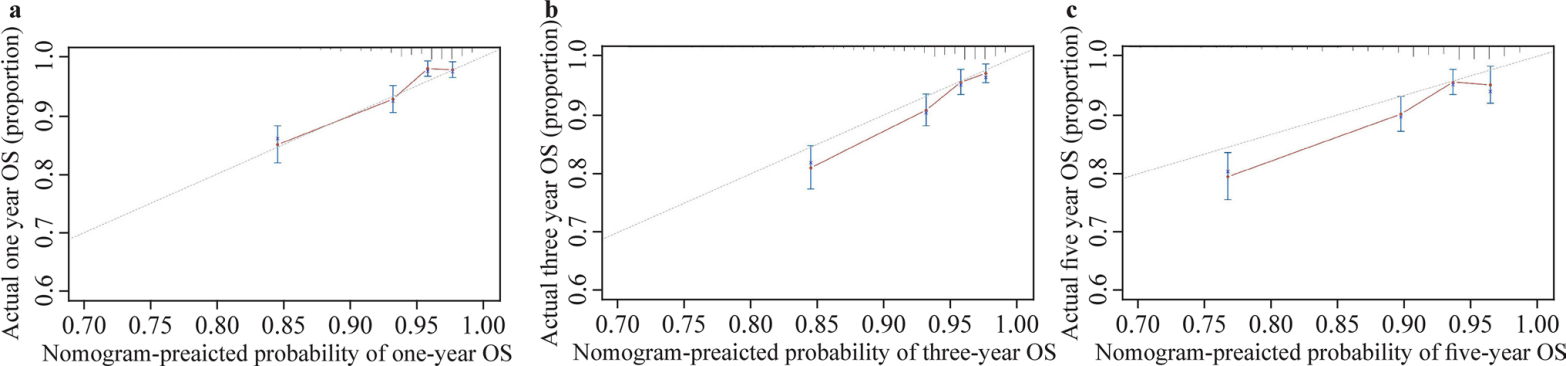
Calibration curves for allograft survival after pediatric liver transplantation scores. Calibration curves showing allograft survival at **a** 1 year, **b** 3 years, and **c** 5 years posttransplantation. The nomogram-predicted probability of OS is plotted on the x-axis, actual OS is plotted on the y-axis. Grey line showed the ideal model where predicted probability fully aligned with the actual probability. *OS* overall survival

The results of internal validation are illustrated by the AUC with the 95% CI and calibration plots. The bootstrap validation exhibited a significant prediction accuracy for both ASPELT and ASPELT/IL-1β. The AUCs for predicting graft survival at 1, 3, and 5 years were 73.27% (95% CI 68.25–77.76%), 67.49% (95% CI 62.27–72.31%), and 60.80% (95% CI 54.87–66.43%), respectively, for ASPELT and 73.44% (95% CI 68.43–77.92%), 67.67% (95% CI 62.58–72.37%), and 61.15% (95% CI 55.25–66.74%), respectively, for ASPELT/IL-1β (Fig. 3a–c). The calibration plots created using bootstrap resampling also suggested that the 1-, 3-, and 5-year predicted survival rates agreed with the actual survival rates (Fig. 4a–c).

### Comparison with other prediction models

**Fig. 5:**
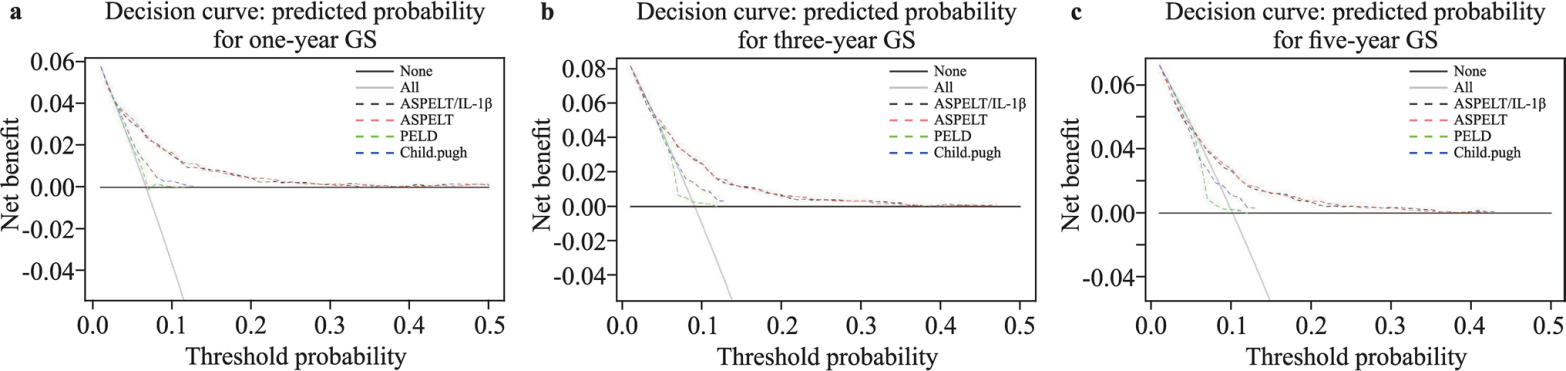
Decision curve analysis for ASPELT. The curves show the analyses at **a** 1 year, **b** 3 years, and **c** 5 years posttransplantation. The y-axis is the net benefit, and the x-axis denotes the threshold probability. *ASPELT* allograft survival after pediatric liver transplantation, *PELD* Pediatric End-Stage Liver Disease, *IL* interleukin, *GS* graft survival

According to the results of the ROC and decision curve analyses, our prediction model outperformed the existing prediction scoring systems, PELD and Child–Pugh, at all three predicting points (Figs. 3a–c and 5a–c). Figure 5a–c indicates that the use of ASPELT and ASPELT/IL-1β had a higher net benefit than the other two models over a wide range of threshold probabilities.

### Clinical use

**Fig. 6:**
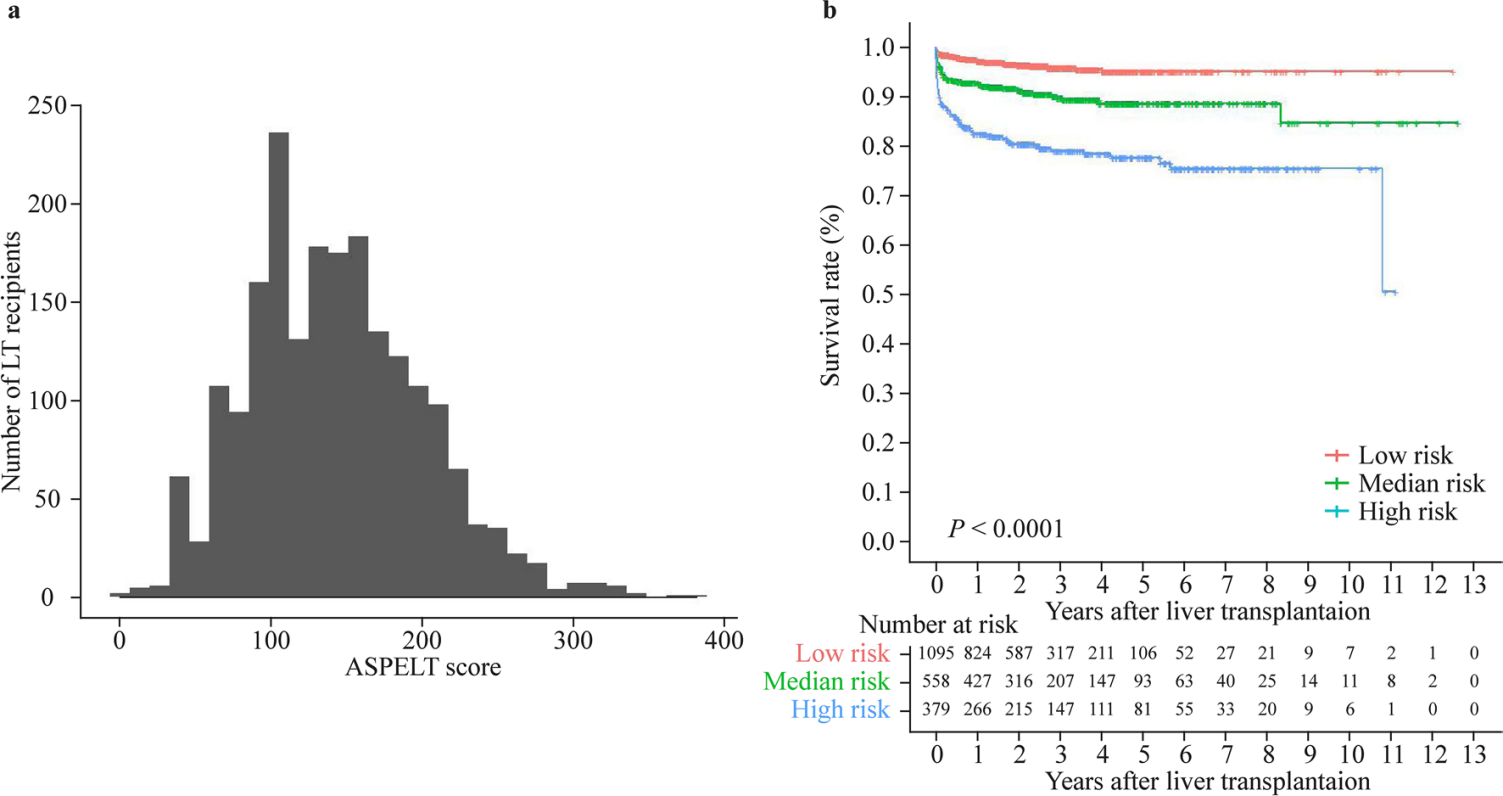
Distribution of ASPELT scores and survival rates based on the ASPELT score. *ASPELT* allograft survival after pediatric liver transplantation, *LT* liver transplantation

The ASPELT scores showed a normal distribution (Fig. 6a). Based on the final score, the cutoff values to determine the risk of poor allograft outcomes were estimated to be at scores of 146 and 196. Recipients with a score below 146 were assigned to the low-risk group, those with a score of 146–196 to the median-risk group, and those with a score above 196 to the high-risk group (Supplementary Table 4). With an increase in risk, the graft survival rate at 1, 3, 5, and 10 years after the operation decreased significantly (*P* < 0.001; Fig. 6b). The ASPELT model can also be accessed on the Internet or via a web application at https://aspelt.shinyapps.io/ASPELT/.

## Discussion

In our study, we established a prognostic tool to enhance the prediction of allograft survival after pediatric LT. The evaluation of the performance of our prediction model, ASPELT, suggested that it can precisely categorize LT recipients into low-risk, median-risk, and high-risk groups, wherein a significant difference in the allograft survival rates was noted among the groups. Furthermore, this prediction model has a higher prediction accuracy than the other models previously used.

Our study determined that the preoperative diagnosis, recipient age, body weight, surgical type, preoperative serum total bilirubin level, serum IL-β level, portal venous blood flow direction, spleen thickness, and presence of heart disease and cholangitis were independent risk factors associated with graft survival in pediatric LT. Therefore, we developed ASPELT, a 10-variable nomogram, to predict graft survival at 1, 3, and 5 years. Based on the ASPELT score, the recipients were divided into high-risk, median-risk, and low-risk groups. With an increase in risk, the graft survival rate at 1, 3, 5, and 10 years after the operation decreased significantly (*P* < 0.0001). As the most widely used tool to evaluate the prognosis of patients with liver disease [14, 15], the PELD and Child–Pugh scoring systems were also compared with ASPELT.

The PELD score was used to determine the priority of pediatric LT candidates based on the risk of death before transplantation [16]. It was developed using bilirubin, INR, serum albumin, age, and growth failure to predict the probability of death on the waiting list. Some studies have shown a correlation between the PELD score and posttransplant survival [15], while others have suggested that the PELD score is not an accurate predictor of the outcomes following transplantation [17, 18]; therefore, the prediction efficiency of PELD has remained undecided. Our study found that the C-indices of PELD for predicting graft survival at 1, 3, and 5 years were 0.548, 0.546, and 0.544, respectively, which were minimally satisfactory.

The Child–Pugh score has been widely used for the assessment of prognosis in liver cirrhosis [19]. In previously reported studies, the variation in survival explained by the Child–Pugh score remains somewhat low (< 50%) [20]. The Child–Pugh score has several limitations [21]. First, it contains subjective variables, such as ascites and encephalopathy, leading to unavoidable deviation in determining the severity of the recipients’ condition. Second, the stratification of the continuous variables also affects the prediction efficiency of the scoring model due to its ceiling or floor effects, which suggests that the Child–Pugh classification may hardly differentiate between patients with an albumin level of 15 g/L versus 24 g/L. In our study, the C-indices of the Child–Pugh scoring system for predicting graft survival at 1, 3, and 5 years were 0.617, 0.618, and 0.615, respectively; these were weaker than those of ASPELT (0.776, 0.757, and 0.755, respectively) and ASPELT/IL-1β (0.774, 0.751, and 0.749, respectively).

Previous prediction models, such as PELD or Child–Pugh, were developed using data either acquired from electronic health records, wherein only preoperative laboratory measurements and physical features were available, or from a single-center study on a small cohort. The ASPELT, however, has been established based on more than 2000 cases, and it considers patient comorbidities, visceral features, and other surgical characteristics apart from those variables used in other models. It also prospectively evaluates the effect of inflammatory responses even though it has not been a regular test before an LT.

The ASPELT score model offers several key advantages in clinical practice. By considering multiple risk factors, the ASPELT score provides a comprehensive assessment of graft survival probability for individual patients. This enables clinicians to develop personalized treatment plans based on each patient’s unique risk profile. Tailored interventions, such as intensified immunosuppressive therapy or alternative treatment strategies, can be implemented, optimizing posttransplant care and improving outcomes. The accurate prediction of graft survival offered by the ASPELT score assists transplant centers in prioritizing candidates based on their individual risk profiles. The ASPELT score allows for precise risk categorization of LT recipients into low-risk, median-risk, and high-risk groups based on preoperative variables. This enables clinicians to identify patients at higher risk of graft failure and tailor their management accordingly. By closely monitoring high-risk patients and adjusting treatment strategies, clinicians can potentially improve graft survival rates and overall patient outcomes.

The limitation of this retrospective study mainly lies in the lack of external validation. If any record from a different transplantation center can be used for model validation, the model performance evaluation can be more robust. Second, this study was conducted at a single center and may, therefore, be weaker in methodology than studies using randomly sampled populations, especially when the prognosis of transplantation may depend on the clinical logistics or responsible surgeon that varies from center to center and is difficult to quantify. Another drawback is the nontraceable missing data, which is inherent in this type of research.

In conclusion, the ASPELT score model can effectively predict the graft survival rate after LT in children, providing a simple and convenient evaluation method for clinicians and patients. The ASPELT score model is not limited to research applications but has direct implications for clinical practice in pediatric LT. By utilizing the ASPELT score, clinicians can enhance patient care, improve outcomes, and contribute to advancements in the field of pediatric liver transplantation.

### Supplementary Information

Below is the link to the electronic supplementary material.

Supplementary file1 (DOC 575 KB)

## Data Availability

The authors confirmed that the data supporting the findings of this study are available within the article and/or supplementary materials.
