# Supplementary material for: Development and validation of a nomogram to predict allograft survival after pediatric liver transplantation
Source: World J Pediatr. 2023 Oct 24;20(3):239–49. doi: 10.1007/s12519-023-00766-y (PMC10957674; doi:10.1007/s12519-023-00766-y)
Supplement: Supplementary file 1 — Supplementary file1 (DOC 575 KB) [file 12519_2023_766_MOESM1_ESM.doc]

**Development and validation of a nomogram to predict allograft survival after pediatric liver transplantation**

**Supplementary Material**

**Supplementary figure legends**

**Supplementary Fig. 1.** **Geographic distribution of children undergoing liver transplantation at Renji Hospital from 2006 to 2019**

**Supplementary Fig. 2. Kaplan–Meier survival curves for each covariate in the ASPELT model, including diagnosis (A), recipient age (B), recipient total bilirubin (C), heart disease (D), surgical method (E), direction of portal vein flow (F), cholangitis (G), recipient spleen thickness (H), and recipient body weight (I).**

The y-axis indicates the graft survival rate and the x-axis indicates the years after liver transplantation. The P-value for each group, determined using the log-rank test, is on the corresponding plot.

**Supplementary tables**

**Supplementary Table 1. Indications for pediatric liver transplantation**

**Supplementary Table 2.The score of each covariate**

**Supplementary Table 3.**  **The C-index for each model of graft survival**

**Supplementary Table 4.**  **Allograft survival rates in the low-, median-, and high-risk groups**


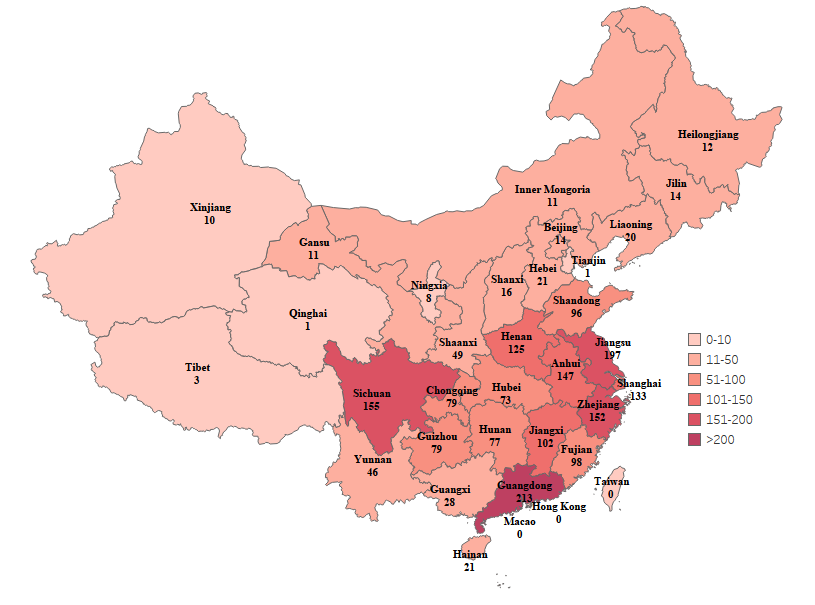


**Supplementary Fig. 1.** **Geographic distribution of children undergoing liver transplantation at Renji Hospital from 2006 to 2019**


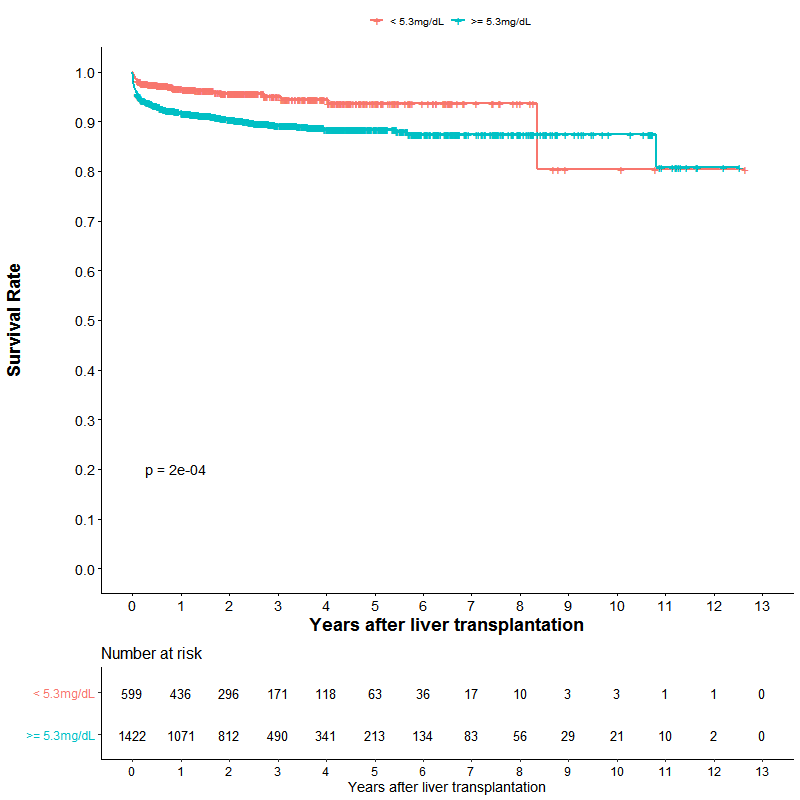

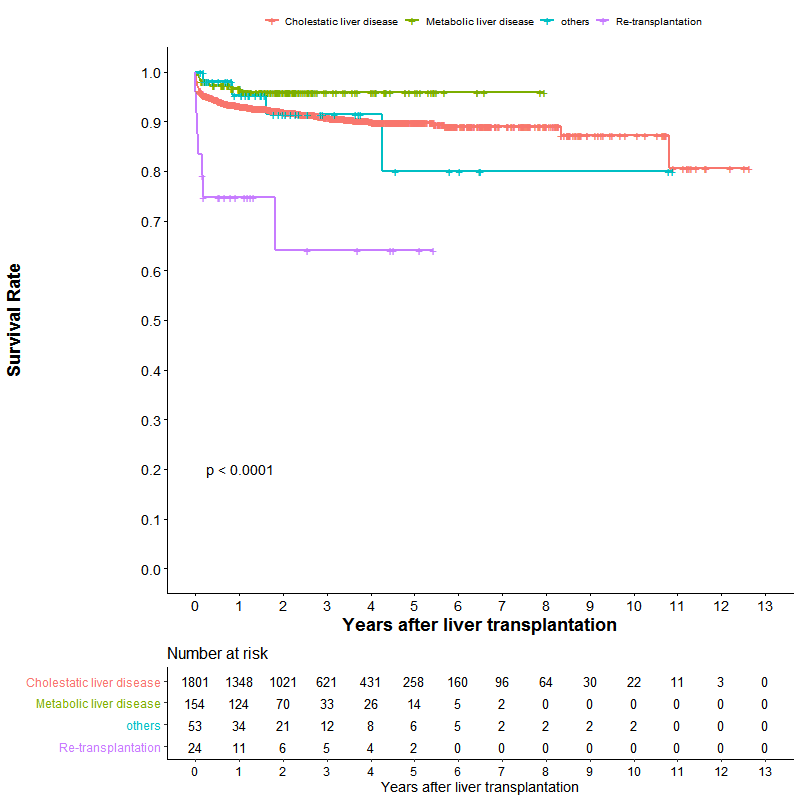

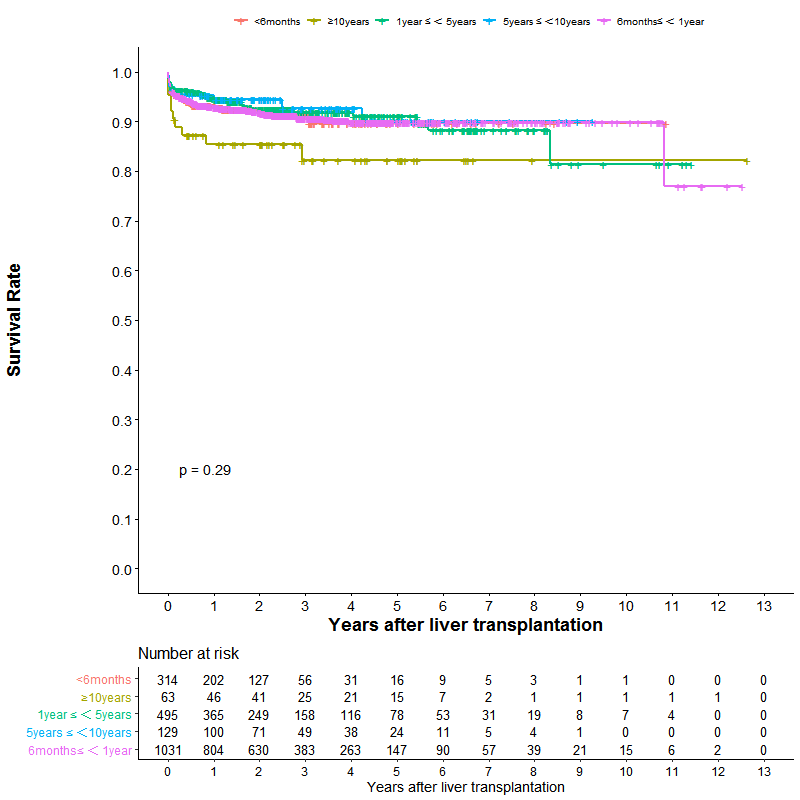

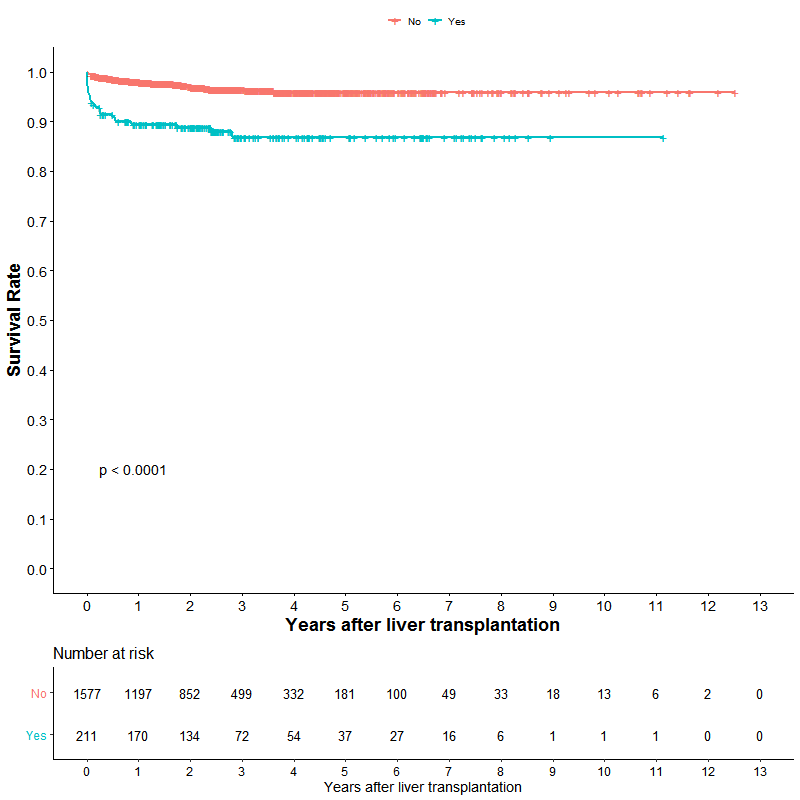

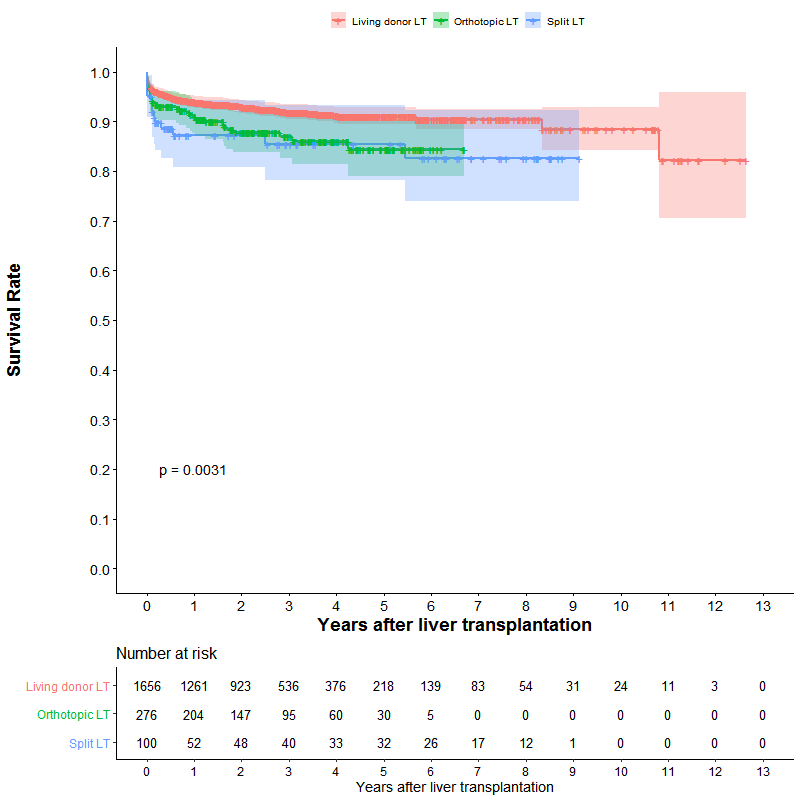

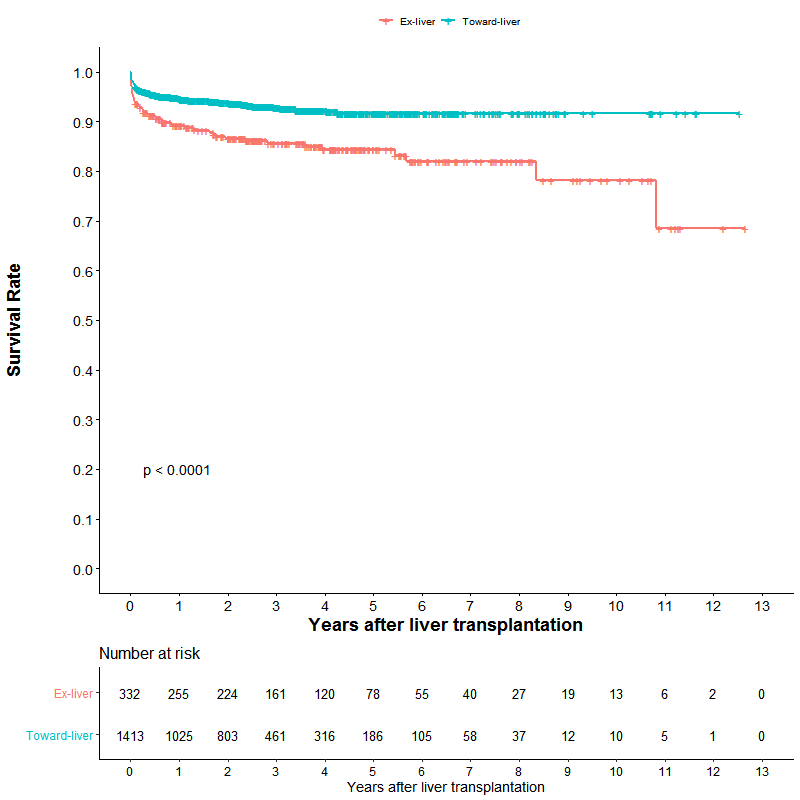

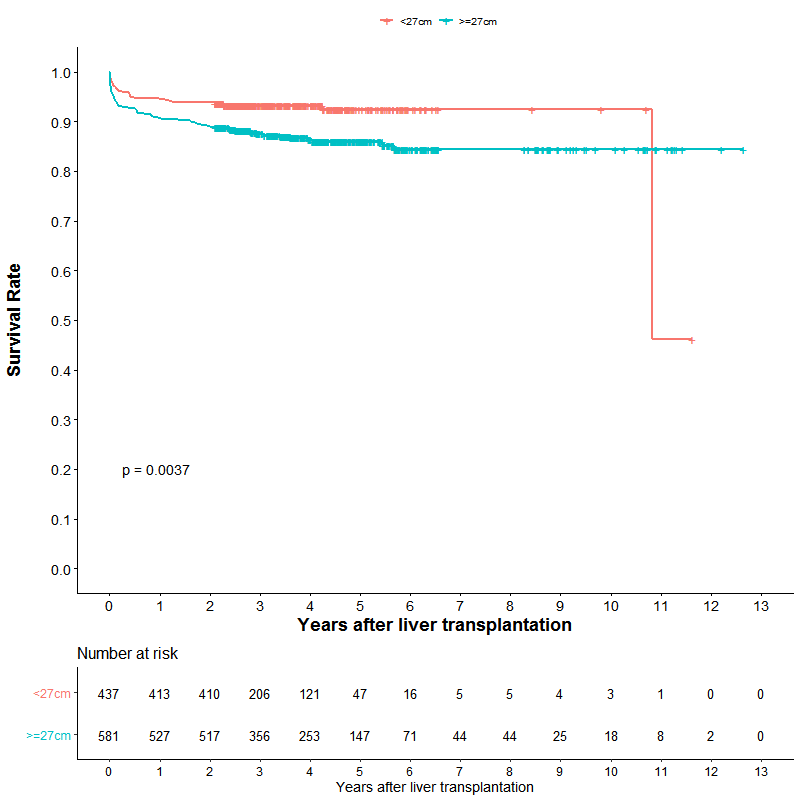

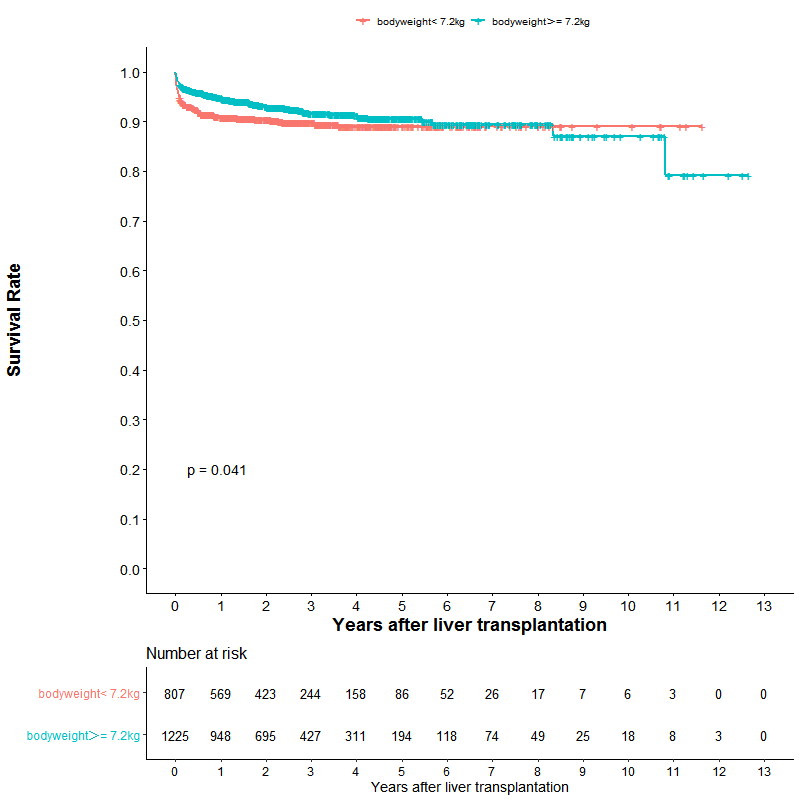

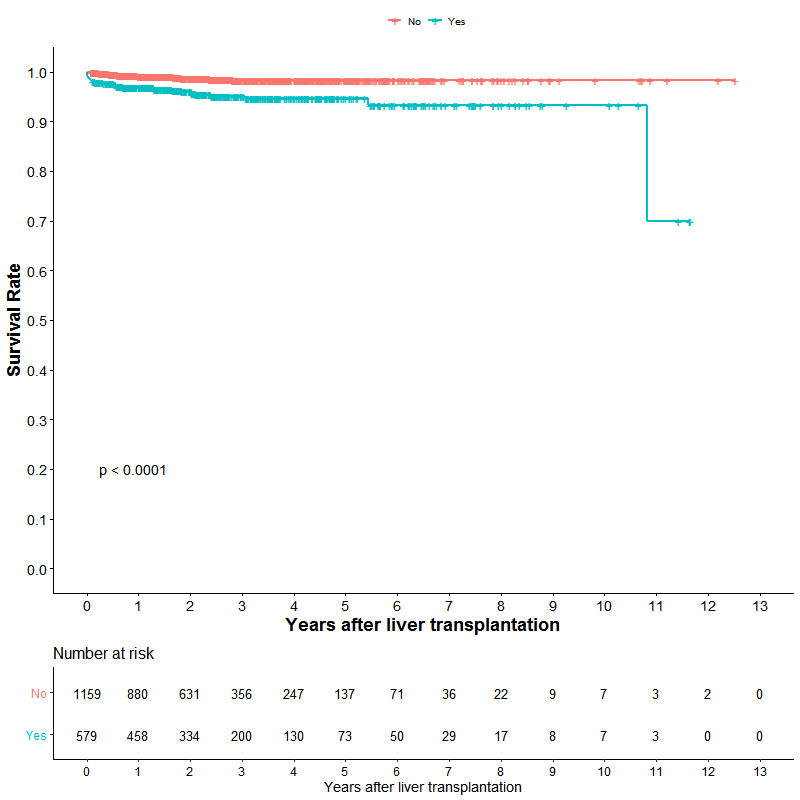

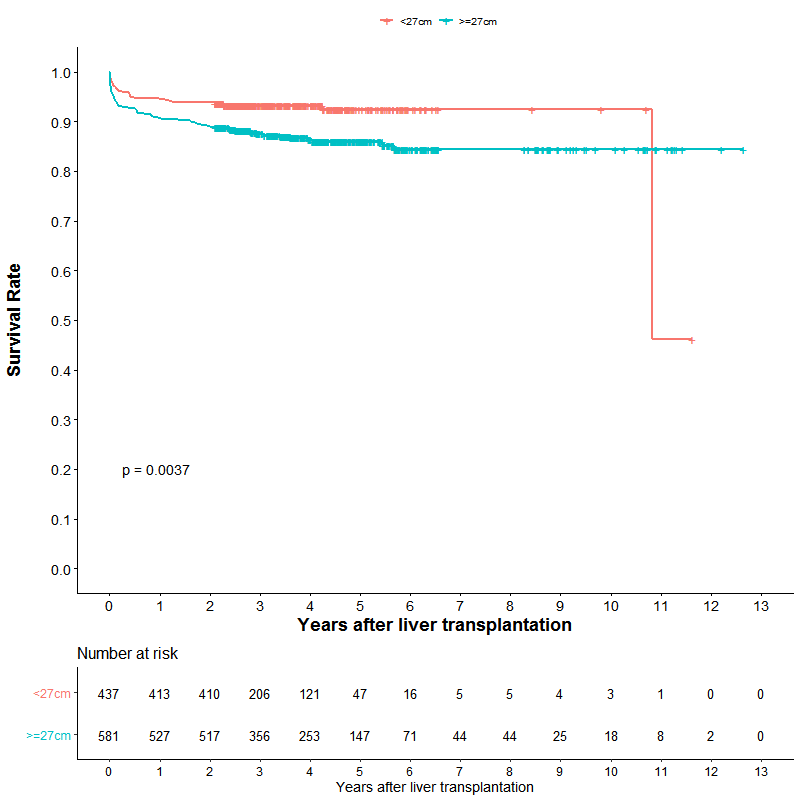


**A**

**B**

**C**

**A**

**B**

**D**

**E**

**F**

**G**

**H**

**I**

**Supplementary Fig. 2. Kaplan–Meier survival curves for each covariate in the ASPELT model, including diagnosis (A), recipient age (B), recipient total bilirubin (C), heart disease (D), surgical method (E), direction of portal vein flow (F), cholangitis (G), recipient spleen thickness (H), and recipient body weight (I).**

The y-axis indicates the graft survival rate and the x-axis indicates the years after liver transplantation. The P-value for each group, determined using the log-rank test, is on the corresponding plot.

| **Supplementary Table 1. Indications for pediatric liver transplantation** | | | |
| --- | --- | --- | --- |
|  | **n** | **%** |  |
| Cholestatic liver disease | 1801 | 88.6 |  |
| Biliary atresia | 1647 | 81.1 |  |
| Progressive familial intrahepatic cholestasis | 40 | 2 |  |
| Cryptogenic cirrhosis | 33 | 1.6 |  |
| Alagille syndrome | 31 | 1.5 |  |
| Cholestasis | 22 | 1.1 |  |
| Caroli disease | 21 | 1 |  |
| Congenital bile acid synthesis defect | 4 | 0.2 |  |
| Congenital biliary dysplasia | 3 | 0.1 |  |
| Metabolic liver disease | 154 | 7.6 |  |
| Methylmalonic academia | 31 | 1.5 |  |
| Ornithine transcarbamylase deficiency | 31 | 1.5 |  |
| Propionic acidemia | 23 | 1.1 |  |
| Tyrosinemia | 14 | 0.7 |  |
| Glycogen storage disease | 13 | 0.6 |  |
| Niemann-Pick Disease | 9 | 0.4 |  |
| Wilson’s disease | 9 | 0.4 |  |
| Citrullineemia | 6 | 0.3 |  |
| Carbamoyl phosphate synthase I deficiency | 4 | 0.2 |  |
| Hypercholesterolemia | 3 | 0.1 |  |
| Argininosuccinic aciduria | 2 | 0.1 |  |
| Citrin Deficiency | 2 | 0.1 |  |
| Hyperoxaluria | 2 | 0.1 |  |
| Alpha-1-antitrypsin deficiency | 1 | 0.0 |  |
| Arginineemia | 1 | 0.0 |  |
| Hyperhomocysteinemia | 1 | 0.0 |  |
| maple syrup urine disease | 1 | 0.0 |  |
| x-linked agammaglobulinemia | 1 | 0.0 |  |
| Neoplastic disease | 28 | 1.4 |  |
| Hepatoblastoma | 17 | 0.8 |  |
| Langerhans histiocytosis | 6 | 0.3 |  |
| hemangioendothelioma of the liver | 3 | 0.1 |  |
| Hepatic hamartoma | 1 | 0.0 |  |
| Hepatoembryonic sarcoma | 1 | 0.0 |  |
| Re-transplantation | 24 | 1.2 |  |
| Acute liver failure | 13 | 0.6 |  |
| Drug-induced liver injury | 3 | 0.1 |  |
| Unknown | 10 | 0.5 |  |
| Vascular disease | 12 | 0.6 |  |
| Cavernous transformation of portal vein | 5 | 0.2 |  |
| Budd-Chiari syndrome | 4 | 0.2 |  |
| Congenital portosystemic shunts | 2 | 0.1 |  |
| telangiectasis | 1 | 0.0 |  |
| Total | 2032 | 100 |  |
|  |  |  |  |

| **Supplementary Table 2.The score of each covariate** | |
| --- | --- |
|  | **score** |
| Diagnosis |  |
| Cholestatic liver disease | 0 |
| Metabolic liver disease | 15 |
| Re-transplantation | 100 |
| Others | 24 |
| Age |  |
| 6 months–1 year | 0 |
| <6months | 11 |
| ≥10years | 80 |
| 1year-5years | 9 |
| 5years-10years | 22 |
| Total Bilirubin |  |
| < 5.3mg/dL | 0 |
| ≥ 5.3mg/dL | 68 |
| Heart Disease |  |
| No | 0 |
| Yes | 60 |
| Surgical method |  |
| Living donor liver transplantation | 0 |
| Orthotopic liver transplantation | 47 |
| Split liver transplantation | 54 |
| Direction of portal vein flow |  |
| Toward-liver | 0 |
| Ex-liver | 50 |
| Cholangitis |  |
| No | 0 |
| Yes | 51 |
| Spleen thickness |  |
| < 27mm | 0 |
| ≥ 27mm | 30 |
| Body Weight |  |
| ≥7.2kg | 0 |
| <7.2kg | 40 |
| IL-1β |  |
| < 7.54 | 0 |
| ≥ 7.54 | 22 |
| Total |  |

Abbreviation: IL, interleukin

| **Supplementary Table 3.**  **The C-index for each model of graft survival** | | | | | | |  |  |
| --- | --- | --- | --- | --- | --- | --- | --- | --- |
| **Criteria** | **ASPELT** | **ASPELT-IL-1β** | **PELD** | **Child-Pugh** | **Pedi-SOFTScore** | **pCLIF-SOFAScore** |  | |
| AIC | 2419.774 | 2420.960 | 2494.006 | 2470.827 | 2486.379 | 2491.985 |  | |
| C-index for 1-year graft survival | 0.776 | 0.774 | 0.548 | 0.617 | 0.560 | 0.564 |  | |
| C-index for 3-year graft survival | 0.757 | 0.751 | 0.546 | 0.618 | 0.554 | 0.558 |  | |
| C-index for 5-year graft survival | 0.753 | 0.749 | 0.544 | 0.615 | 0.553 | 0.556 |  | |

Abbreviations: ASPELT, allograft survival after pediatric liver transplantation; PELD, pediatric end-stage liver disease; AIC, Akaike information criterion; IL, interleukin

| **Supplementary Table 4.**  **Allograft survival rates in the low-, median-, and high-risk groups** | | | | | | |
| --- | --- | --- | --- | --- | --- | --- |
| Group | n | % | Survival Rate(%) | | | |
|  |  |  | 1 year | 3 year | 5 year | 10 year |
| Low Risk（ASPELT score <146） | 1095 | 53.9 | 97.4 | 96 | 95.1 | 95.1 |
| Median Risk（ASPELT score 146≤ <196） | 558 | 27.5 | 92.7 | 89.7 | 88.7 | 84.7 |
| High Risk（ASPELT score ≥196） | 379 | 18.6 | 82.4 | 79 | 77.6 | 75.3 |

Abbreviation: ASPELT, allograft survival after pediatric liver transplantation
